# Alginic Acid Beads Containing Fluorescent Solvatochromic Dyes Display an Emission Color Response to a Cationic Surfactant

**DOI:** 10.3390/polym14214649

**Published:** 2022-11-01

**Authors:** Kazuki Kishi, Amane Ichimura, Zhang Shuai, Yu Otsuka, Tatsuya Morozumi, Koji Yamada

**Affiliations:** 1 Division of Materials Science, Graduate School of Environmental Science, Hokkaido University, Sapporo 060-0810, Hokkaido, Japan; 2 Division of Environ Science, Graduate School of Environmental Science, Hokkaido University, Sapporo 060-0810, Hokkaido, Japan; 3 Hokkaido Institute of Public Health, Sapporo 060-0819, Hokkaido, Japan; 4 Division of Materials Science, Faculty of Environmental Earth Science, Hokkaido University, Sapporo 060-0810, Hokkaido, Japan

**Keywords:** fluorescent solvatochromic dye, alginate gel, cetylpyridinium chloride, adsorption, fluorescence microscopy image, hue analysis, quantitative measurement

## Abstract

Lipophilic fluorescent dyes can be employed as sensors for surfactants present in concentrations above the critical micellar concentration (CMC) where the dyes are monodispersed in micelles. However, the surfactant concentration range over which these dyes are effective is narrowed because by the sigmoidal nature of their responses. To overcome this limitation, we developed a novel sensor material comprised of a labeled fluorescent solvatochromic dye covalently bonded to alginate gel, which is known to strongly adsorb cationic surfactants. We hypothesized that the dye-alginate conjugate would undergo fluorescent color changes in response to binding of surfactants which alter the polarity of the surrounding environment. Indeed, addition of the representative cationic surfactant, cetylpyridinium chloride (CPC), to an aqueous solution of the alginate conjugated fluorescent solvatochromic dye leads to a visible fluorescent color change when the concentration of CPC is below the CMC. The average values of the color appearance parameter, referred to as a hue, of light emitted from gels, calculated by analysis of fluorescence microscopy images using ImageJ software, were found to be approximately linearly dependent on the concentration of CPC encapsulated in the alginate-fluorescent dye complex. This finding shows that absorbed CPC can be quantitatively determined over a wide concentration range in the form of simple fluorescence wavelength or visible responses.

## 1. Introduction

Fluorescent solvatochromic dyes have emission colors that depend on the nature of the solvent [1,2,3,4,5,6,7,8,9]. In homogeneous solutions, the emission wavelength maxima of these dyes are linearly dependent on the solvent polarity parameter E_T_(30) [5,6]. The emission wavelength maxima also change when the solvatochromic dyes are components of heterogeneous systems containing aggregates of amphipathic molecules that contain both polar and non-polar sites. For example, Son et al. devised an interesting amphipathic fluorescent solvatochromic dye that is comprised of a highly polar electron-withdrawing pyridinium moiety and a non-polar long-alkyl chain as an electron-donating group. Studies by this group demonstrated that the fluorescence maximum of this dye undergoes large changes when present in solutions containing micelle forming surfactants such as cetyltrimethylammonium bromide (CTAB) [1]. Because this pyridinium dye undergoes an emission change that is dependent on the type of surfactant, its solvatochromic behavior is likely governed by the polarity of the environment present in the surfactant dye complex. However, because the fluorescence color changes take place in a sigmoidal manner around the critical micelle concentration (CMC) of the surfactant, the dye is not suitable for quantitative determination of surfactant concentrations.

In the context of sensor applications, typical solvatochromic dyes have a disadvantageous feature associated with fluorescence self-quenching that takes place upon aggregate formation in solvents of low solubility. To prevent self-quenching, Otsuka et al. previously developed a sensing material in which a fluorescent solvatochromic dye is immobilized on the surface of polystyrene [2]. Importantly, we found that when this dye-polymer complex is immersed in solvents with different polarities, solvent dependent fluorescence color changes occur, with a linear correlation exiting between E_T_(30) values and fluorescence wavelengths.

**Figure 1:**
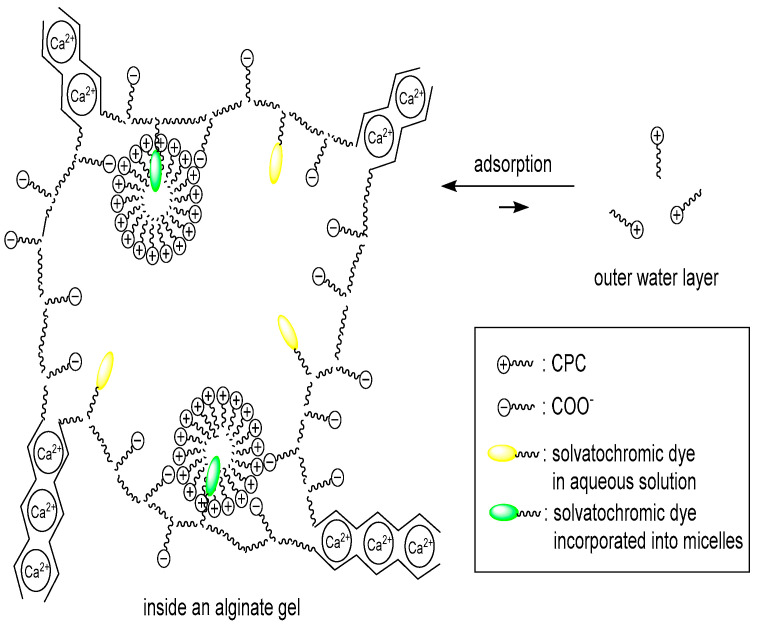
The proposed process involved in CPC detection using tan alginate gel linked fluorescent solvatochromic dye.

These observations, which show that fluorescent solvatochromic dyes are excellent sensors even when located at heterogeneous interfaces such as the polymer surfaces, stimulated an investigation aimed at exploring new sensor systems in which fluorescent solvatochromic dyes are covalently bound to polymers that readily absorb surfactants in aqueous solutions. The criterion we used to select an appropriate polymer include ready availability, low cost, an ability to undergo gelation using a simple operation and the presence of substituents that facilitate immobilization of fluorescent solvatochromic dyes. These considerations led to identification of alginate as an appropriate polymer. Alginic acid, a polysaccharide found in brown algae such as wakame seaweed, is commercially available in a variety of molecular weight ranges. Moreover, addition of multivalent ions such as Ca^2+^ to aqueous solutions of sodium alginate generates a well studied alginate gel [10,11,12,13,14,15,16,17,18,19,20,21,22,23,24]. The alginate prepared in this way contains numerous free carboxylic acid groups that are not involved in crosslinking with multivalent ions and, thus, can be utilized for amide bond formation with a substance containing a terminal primary amine moiety. Thus, we envisioned that it might be possible to link a terminal amine containing fluorescent solvatochromic dye to the alginate gel by amide bond formation [14]. We also hypothesized that a surfactant would be effectively absorbed by charge-charge interactions with the remaining free carboxylate groups in the alginate-dye conjugate (Figure 1) [14,24]. Furthermore, we anticipated that surfactant binding to fluorescent solvatochromic dye containing gel would alter the polarity of the environment. Consequently, the dye in the surfactant bound complex would display a characteristic fluorescent wavelength response to a surfactant at concentrations below the CMC.

In the investigation described below, we prepared the new terminal amine containing fluorescent solvatochromic dye **10** and transformed it to the alginate bonded derivative **12**. In addition, we evaluated the use of **12** as a florescence sensor for quantitative determination of the concentration of the surfactant cetylpyridinium chloride (CPC). Finally, with the aim of developing a sensor that does not require use of a fluorescence spectrometer, we explored a quantification protocol that is based on fluorescence image analysis.

We also hypothesized that a surfactant would be effectively absorbed by charge-charge interactions with the remaining free carboxylate groups in the alginate-dye conjugate. As the local concentration of CPC inside the gel increased and exceeded CMC, it may be to form stable micelles. It is thought that the hydrophobic fluorescent solvatochromic dye labeled on the gel may aggregate in the hydrophobic core of that micelle. Consequently, the dye in the surfactant bound complex would display a characteristic fluorescent wavelength response to a surfactant at concentrations below CMC.

## 2. Materials and Methods

### 2.1. Reagents and Instruments

All the reagents were purchased from Wako Pure Chemical and used without further purification. Nuclear magnetic resonance (NMR) spectra were recorded using a JNM-EX400 manufactured by Japan Electronics Co., Ltd. (Tokyo), trimethylsilyl (TMS) was used as a reference. Mass spectrometry was performed by the Instrumental Analysis Division, Global Facility Center, Creative Research Institution, and Hokkaido University. ESI-LH was measured using a Thermo Scientific™ Exactive™ Plus instrument. Attenuated total reflection infrared (ATR-IR) spectra were recorded using a Thermo Fisher Scientific NicoletiS10FT-IR spectrometer with GladiATRTM accessories. Absorption spectra were obtained using a JASCO V-770 spectrophotometer. Microscope observations were carried out using an Olympus research high-grade stereoscope SZX16 or the Olympus inverted fluorescence microscope IX70-S1F.

### 2.2. Synthesis

**Preparation of alginate gel beads (11)**

Alginate gel beads were prepared using the procedure given in Reference [25]. Specifically, 200 mL of an aqueous solution of sodium alginate (2 g corresponding to 0.01 mol of carboxylic acid groups) was added dropwise to a CaCl_2_ aqueous solution (20 g, 0.18 mol, 200 mL). The generated material was thoroughly washed with water and stored in water. A defined portion of the material was dried for 24 h and weighed. The result provided an average dry weight for the produced alginate gel beads (**11**) of 3.56 mg (9.1 µmol of carboxyl acid groups obtained from reference [25]).

**Preparation of Alginate Linked Fluorescent Solvatochromic Dye (12)**

1-Ethyl-3-(3-dimethylaminopropyl)carbodiimide hydrochloride (WSC) (2.5 eq, 2.5 mmol, 0.44 g) was added to five independent mixtures of alginate gel beads (dry weight 3.6 g, 9.1 mmol, 100 beads) in 10 mL of mixed solution of water and dimethylformamide(DMF) (1:1) at 5 °C. After stirring at 100 rpm for 30 min, an aqueous HOBt solution of 1-hydroxybenzotriazole monohydrate (HOBt·H_2_O) (1.35 eq, 1.2 mmol, 0.17 g) was added to each mixture at 5 °C followed by stirring at 100 rpm for 30 min. To these mixtures were added 0.50, 5.0, 10, 25, and 50 molar equivalents of **10** and the resulting mixtures, were stirred at 30 °C and 100 rpm. The five alginate linked fluorescent solvatochromic dyes **12** produced in this manner, designated as **12a–e**, were thoroughly washed with water and acetone, and then with dichloromethane for 24 h using a Soxhlet extractor. The beads were stored in water.

### 2.3. CPC Adsorption Experiments

#### 2.3.1. Concentration Dependent Adsorption of CPC

CPC adsorption experiments were performed using **12a** and **12b** (5 beads) at 30 °C, 48 h and 100 rpm. To independent aliquots of these solutions were added various concentrations of CPC in the 0–1000 µM range. The bead samples were isolated, lightly washed with water and stored in water. The beads were then photographed using a SZX16-Olympus stereoscopic microscope and 375 nm excitation, and the images were analyzed (HSV format).

#### 2.3.2. Time Dependent Adsorption of CPC

To aqueous solutions of **12a** or **12b** (5 beads) were added 10 mL of 200 µM CPC. The absorbances of these solutions, stirred at 30 °C and 100 rpm, were determined over a 0 to 10 min range.

#### 2.3.3. Batch Type Adsorption of CPC

Independent aqueous solutions of CPC (400 µM, 3.0 mL) containing **12a** and **12b** (10 beads) were monitored over a 13 h period using an absorptiometer. At the same time, aqueous solutions containing **12a** and **12b** (1 bead) and CPC (400 µM, 0.30 mL), were imaged using an Olympus inverted fluorescence microscope IX70-S1F, and the images were analyzed (HSV format). See 2.4 for details of the of image analysis method.

### 2.4. Image Analysis

The captured images saved in jpeg format were converted to the HSV format using the open source software ImageJ (https://imagej.nih.gov/ij/download.html, accessed on 8 October 2022), and the Type”→“HSB stack method”. Next, analyze based on the value of “List” obtained by “Analyze”→“Histogram”. The “list” was created by the following: (1) Cut out 300 × 300 pixels of the image, (2) Get the value of “List” obtained by “Analysis”→“Histogram”, (3) the average value at the obtained values was calculated and analyzed. And specific calculation method is as follows. (1) Multiply the hue value obtained from the list by its number of pixels, (2) Divide the sum of all by 90,000 (=300 × 300). Since the hue values are in the HSV format, the strongest color selected when the saturation is lowered is represented by a numerical value from 0 to 255. For example, hue values are 0 or 255 for red, 42 for yellow, 82 for green, and 170 for blue.

## 3. Results

### 3.1. Preparation of 12

The fluorescent solvatochromic dye **10** used to construct the alginate complexes having differing dye contents contains an electron-acceptor dyad comprised of a respective piperazine nitrogen and 2-acetylthiophene moieties. The electron-donating group and immobilization site nitrogens in **10** are separated by ethylene bridges. As a result, immobilization should not significantly alter the fluorescence properties of the donor-acceptor dyad. In addition, the solubility was good, the connection of the immobilization site was only one substitution, and the yield up **10** was generally good.

**Scheme 1:**
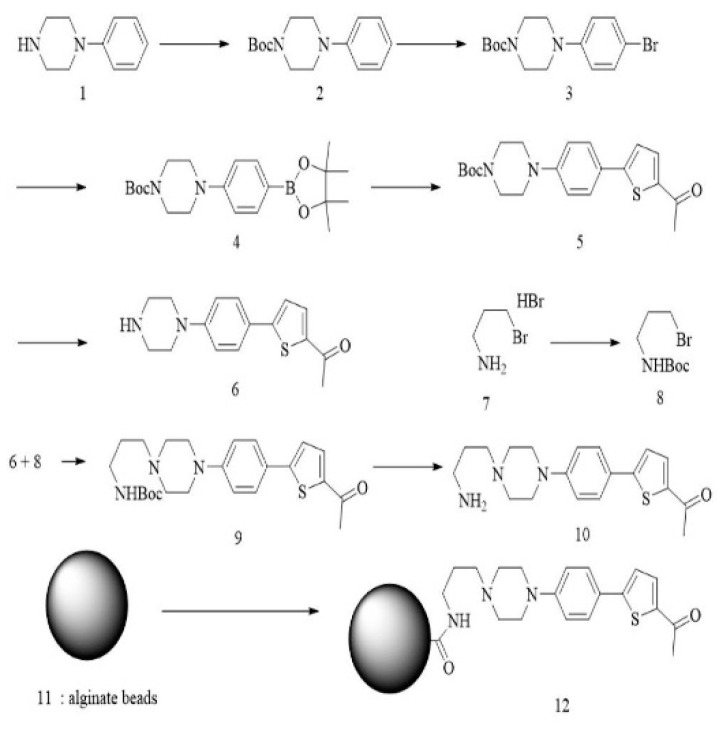
Synthesis of fluorescent solvatochromic dye **10** and alginate linked dye **12**.

The sequence given in Scheme 1 was utilized to synthesize alginate conjugates **12a–e** prepared using differing amounts fluorescent solvatochromic dyes **10**. In the route, *N-*phenylpiperazine (**1**) was Boc protected to generate **2**, which was then transformed to the bromophenyl derivative **3**. Conversion of **3** to boronate **4** was followed by Suzuki-Miyaura cross-coupling with 2-acetyl-5-bromothiophene to form **5**. Removal of the Boc group in **5** generated amine **6**, which was then aminoethylated with 4-*N-*Boc-amino-1-bromoethane (**8** derived from amine **7**) to form **9**, which upon Boc-deprotetion produced the terminal primary amine containing fluorescent solvatochromic dye **10**. Finally, amide bond forming reactions of different quantities of **10** with alginate beads produced the target alginate linked fluorescent solvatochromic dyes **12a–e**.

All substances in the about sequence except **12a–e** were characterized by using ^1^H-NMR, ^13^C-NMR spectroscopy, and mass spectrometry. The detailed synthetic procedures and spectroscopic data are given in Supporting Information Chapter 1, “Synthesis”.

**Figure 2:**
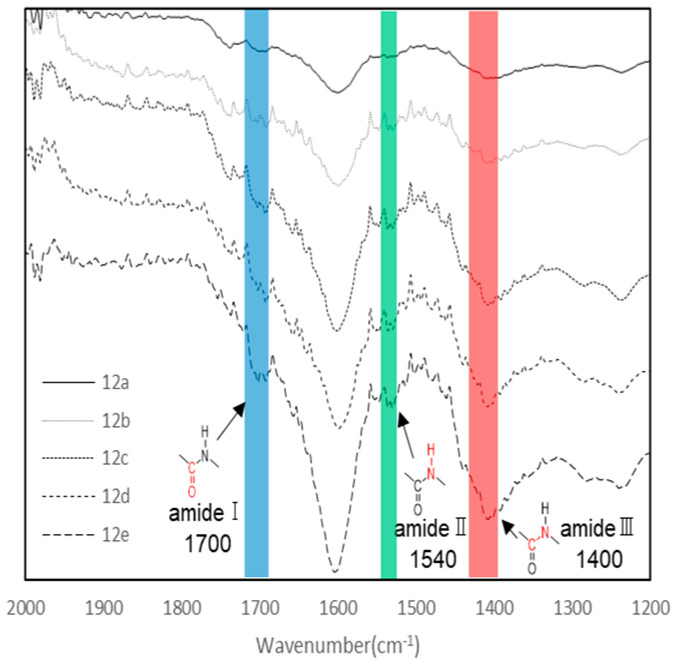
FT−IR spectra of **12a−e**.

Because methods such as NMR cannot be used, we employed IR spectroscopy to confirm that amide bonds are present in **12**. In Figure 2 are displayed portions of the spectra of **12a–e** (see Figures S12 and S13 at Supporting Information for full spectra), which demonstrate that the intensity of amide bands increases as the amount of the fluorescent solvatochromic dye increases, showing that the urea product derived by reaction of WSC is not present in these dye conjugates.

**Figure 3:**
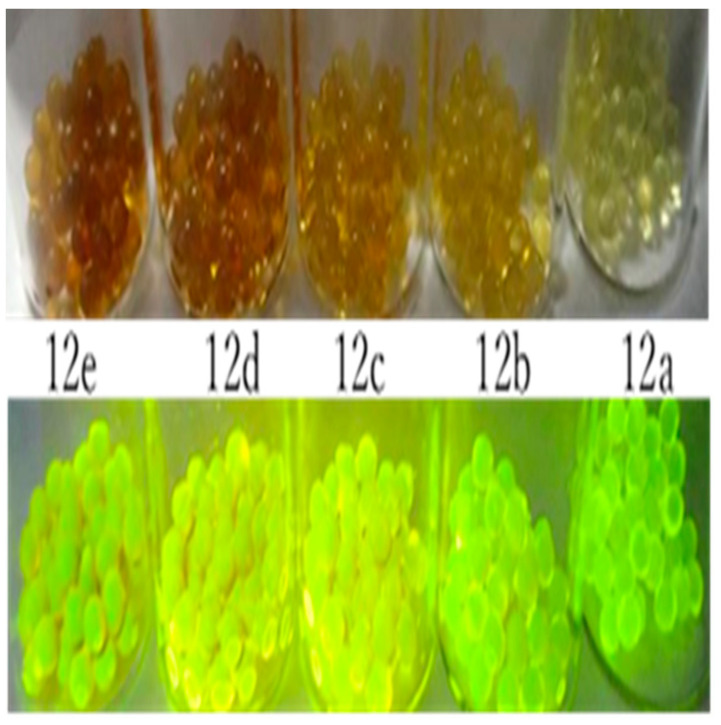
Images of **12a–e** (Top: White light irradiation, Bottom: UV light irradiation).

Analysis of the photographic images of **12a–e** given in Figure 3 shows that **12c** displays the maximum fluorescence intensity. However, because self-quenching operates in **12d,e** to decrease the fluorescence intensity, we reasoned that it also might govern the emission properties of **12c**. As a result, we selected **12a** and **12b** for the studies described below.

### 3.2. Concentration Dependent Adsorption of CPC

**Figure 4:**
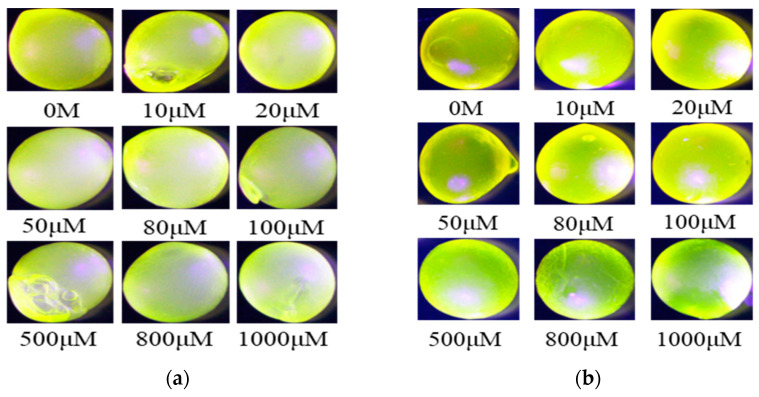
FB and state under UV light irradiation by concentration [(**a**) **12a**, (**b**) **12b**].

**Figure 5:**
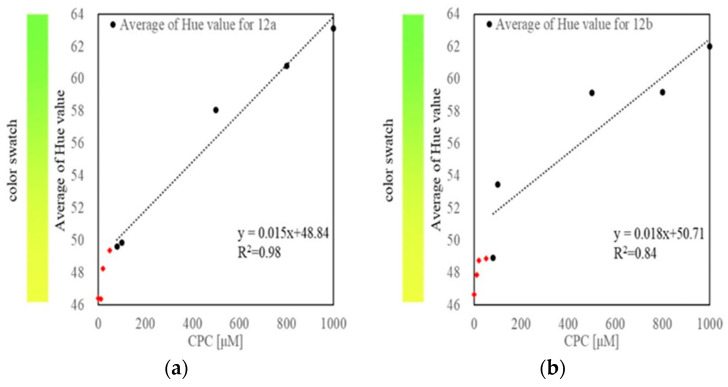
Plots of average hue values of **12a** and **12b** vs. CPC concentration [(**a**) **12a**, (**b**) **12b**]. However, 🔶 is not included in the approximate straight line in Figure 4a,b.

To demonstrate that light emission from **12a,b** responds to CPC, experiments were carried out to determine fluorescence color appearance parameters termed hue values of aqueous solutions of these dye conjugates in the presence of various concentrations of the CPC. The resulting data are provided in Table 1 and Figure 4 and Figure 5.

As can be seen by viewing the data, the presence of increasing concentrations of CPC in aqueous solutions containing these dye-conjugates causes an increase in hue values which reach values of about 62–63 at 1 mM CPC. At the same time, the fluorescence emission colors of the dyes change from lime (about 580 nm) to green-yellow (about 540 nm), and the wavelength maximum undergoes a blue shift. Both phenomena are likely the result of CPC absorption to the alginate complex, which causes a reduction in the polarity of the environment surrounding the covalently bonded fluorescent solvatochromic dye.

Analysis of the plot in Figure 5 of the data for 12a shows that the standard deviation is 0.23 and that of the plot corresponding to **12b** is 0.36. In addition, the slopes of these plots in the 0–50 µM range can be used to show that the lower limits for quantification of CPC concentration are 0.035 µM for **12a** and 0.068 µM for **12b** (See Figures S14 and S15 at Supporting Information).

The above findings, particularly the existence of a correlation between surfactant concentration and hue values, show that **12a,b** are good sensors for CPC. Because the CMC of CPC is in the 900 µM range, the results suggest that **12a,b** respond quantitatively to CPC concentration even when micelles are present in the aqueous solution [26].

However, as described in the introduction, it is thought that there is a change in the adsorption mechanism between the low concentration (0–50 µM) and high concentration (80 µM) of CPC aqueous solution.

In other words, at low concentrations, the CPC molecule approaches the carbonyl group of alginic acid by electrostatic interaction. And if the carbonyl group to which the fluorescent solvatochromic dye is introduced exists in the vicinity, it responds to the fluorescence wavelength.

On the other hand, at high concentrations, the CPC concentration increases in beads and it is caused more CPCs micelles.

Since it is conceivable that quantitative changes could be observed because the amount of fluorescent dye taken into the micelles increased.

### 3.3. Time Depent Addsorption of CPC

To investigate the nature of the sensing process, we determined the rates of adsorption of CPC to the alginate-dye complexes **12a,b** and, for comparison purposes, the dye free alginate **11**. (See Figure S16 at Supporting Information).

**Figure 6:**
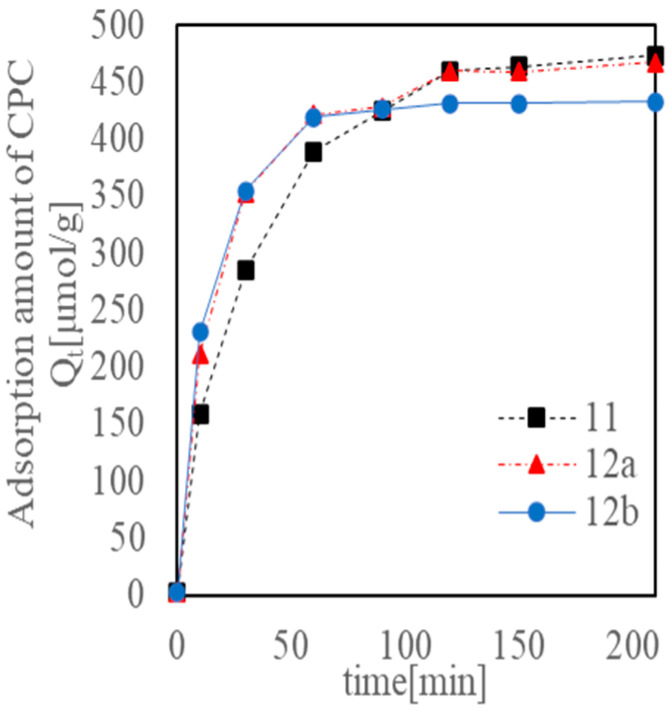
Time dependent adsorption of CPC monitored at 255 nm, the absorption wavelength of CPC on **12a,b** and **11**.

The results (Figure 6) show that the amount of CPC adsorbed per unit time in the 0–90 min range is larger for **12a,b** than for **11**. The difference is thought to be that the CPC was more likely to approach the carbonyl groups in the beads. In this paper, FB were produced by using mixed solution of water and DMF. Because of it, alginate beads might be swelling due to hydrophobic action of DMF. And, alginate beads are left swollen because the fluorescent solvatochromic dye is hydrophobic. It is reasonable that the CPC would reach the carbonyl groups inside more easily than the simple alginate beads, the speed at the initial sage was faster. In addition, the respective amounts CPC adsorbed by **12b** are 41 µmol/g and 34 µmol/g lower than those of **11** and **12a**. This difference is also a consequence of the lower number of carboxylate sites in the latter substances.

### 3.4. Batch Type Adsorption of CPC and Performance Comparison

**Figure 7:**
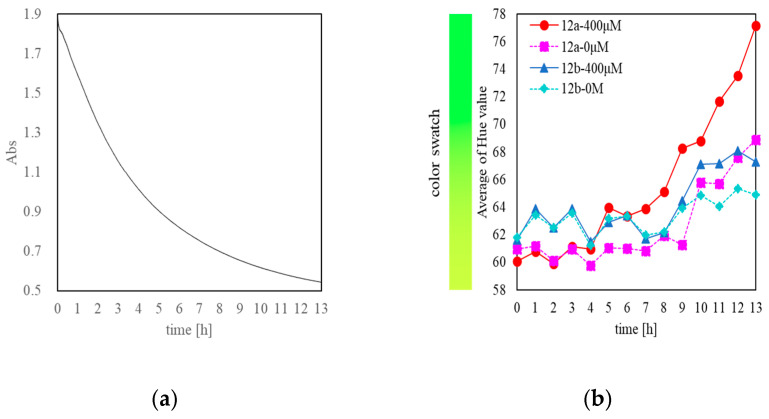
(**a**) Time dependent change of the absorbance of aqueous CPC containing **12a** (10 beads) and (**b**) and time dependent change of average hue values of **12a** and **12b** in batch type.

Finally, a batch type CPC adsorption experiment was carried out using **12a** and **12b**. The results arising from studies are displayed in Figure 7 in the form of plots reflecting the time dependent change of the absorbance of aqueous CPC containing **12a** (10 beads) and (b) of average hue of **12a** and **12b** in batch type systems.

The result of these experiments confirm that adsorption can also be performed in batch type by adding fluorescent beads.

However, regarding shaking time to reach the equilibrium state is 2 h in the non-batch measurements, but in the batch type measurements took 13 h were required to reach equilibrium. In addition, during 2h-shaking time used in the non-batch measurements, almost all of the CPC dissolves in the aqueous solution up to concentration of 1000 µM, but in the batch type measurements, CPCs remains in the solution about 64 µM. Since alginic acid gel having strong mechanical strength is used, use of the shaking type has an advantage when using fluorescent beads produced by this system.

**Figure 8:**
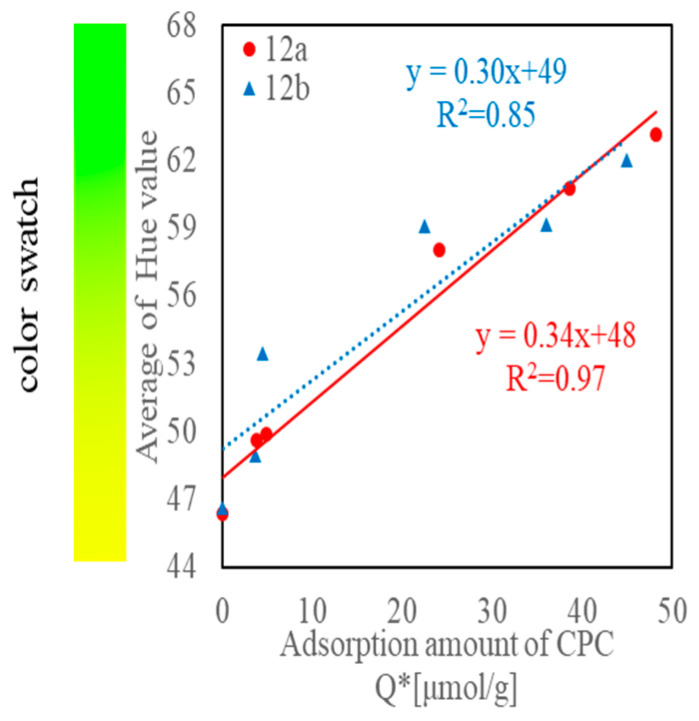
Average hue values and CPC adsorption of **12a,b**.

Next, the relationship between the amount of CPC adsorbed and the average hue value of the **12a,b** was determined (Figure 8).

The result show that a certain correlation in the exists in the adsorption amount range of 0–50 µmol/g, and that a quantitative measurement of the amount absorbed is possible.

However, it was found that **12a** can sensor because it provides more accurate quantification and has a lower limit of quantification. Therefore, the use of **12a** is comparable that of other alginic acid bead sensors given in Table 2.

The data in Table 2. show that the lower limit of detection in this study using fluorescence monitoring, is lower than those of the other systems described in the [27,28,29], which use colorimetric image analysis. This difference is thought to be due to the fact that the fluorescence method tends to have higher detectability than the colorimetric method. In addition, since the chronic toxicity in water of CPC discharged into the environment is 0.56 mM, the fluorescent bead sensor, which has a detection concentration range of 0.035–1.0 mM, is applicable [30].

## 4. Conclusions

In this study, we prepared the novel fluorescent solvatochromic dye **10** and covalently bonded it to an alginate gel. Investigations with this alginate-dye complex show that the alginate-dye complex emits brilliant fluorescence is even when it presents in aqueous solution. This phenomenon is a result of immobilization on the alginate water-soluble gel that prevents aggregation promoted self-quenching. We also demonstrated that the dye complex can be employed for quantitative determination of concentrations of the surfactant CPC in the 0–900 µM range through visually observable fluorescence color changes. The blue-shift in the wavelength of emission of the alginate-dye complex promoted by CPC is thought to be due to absorption of the surfactant that leads to creation of a hydrophobic environment around the fluorescent solvatochromic dye.

Fluorescence microscope images of the dye containing alginate gels confirmed that the average Hue values calculated from the ImageJ software were significantly different depending on CPC concentration. Hue value is linearly approximated by the ratio of the fluorescent solvatochromic dyes incorporated into the micelles to those not incorporated. Therefore, it was found that CPC concentration can be quantitatively measured over a wide concentration range.

Observations made in this study suggest that it should be possible to design novel sensors for a variety of surfactants and biologically relevant substances by incoporating appropriate recognition sites into alginate gel.

## Figures and Tables

**Table 1:** Hue values of **12a,b** in aqueous solutions containing different concentrations of CPC.

CPC Concentration [µM] in Aqueous Solutions		0	10	20	50	80	100	500	800	1000
Average of hue values	**12a**	46.39	46.36	48.26	49.37	49.61	49.86	58.07	60.79	63.14
	**12b**	46.65	47.89	48.75	48.94	48.94	53.47	59.12	59.19	62.01

**Table 2:** Comparison of alginic acid bead mixtures using image analysis.

	Bead Component (Main Component)	M^2+^ Addition Amount	Target	Detection Method	Detection Range [mM]	Detection Lower Bound [μM]	R^2^
This paper **12a**	Alginate— fluorescent solvatochromic dye	CaCl_2_ (0.9 [M])	CPC	Image analysis (Fluorescent)	0.070~1.0	70	0.97
[27]	Alginate— Lactic acid oxidase	CaCl_2_ (400 [mM])	lactic acid	Image analysis (Light absorption)	10~100	6400	0.99
[28]	alginate-methylcellulose— bromothymol blue	CaCl_2_ (0.9 [M])	total volatile basic nitrogen	Image analysis (Light absorption)	344~880	3.4 × 10^5^	0.98
[29]	Alginate— TiO_2_ Nanotubes	TiO_2_—Nanotubes (5 [mg])	lactic acid	Image analysis (Light absorption)	0.10~1.0	230	0.98
			glucose		0.10~0.80	150	0.99

## Data Availability

Not applicable.

## Supplementary Materials

The following are available online at https://www.mdpi.com/article/10.3390/polym14214649/s1, synthesis method of **2**, **3**, **4**, **5**, **6**, **8**, **9** and **10** [31,32], Figure S1: ^1^HNMR spectrum of **5** in CDCl_3_, Figure S2: ^13^C NMR spectrum of **5** in CDCl_3_, Figure S3. ESI-MS (*m*/*z*) of **5**, Figure S4: ^1^HNMR spectrum of **6** in CDCl_3_, Figure S5: ^13^C NMR spectrum of **5** in DMSO, Figure S6. ESI-MS (*m*/*z*) of **6**, Figure S7. ^1^H NMR spectrum of **9** in CDCl_3_, Figure S8: ^13^C NMR spectrum of **9** in CDCl_3_, Figure S9. ESI-MS (*m*/*z*) of **9**, Figure S10: ^1^H NMR spectrum of **10** in CDCl_3_, Figure S11. ESI-MS (*m*/*z*) of **10**, Figure S12. FT-IR spectra of (a) **11**, (b) **12a**, (c) **12b**, (d) **12c**, (e) **12d**, and (f) **12e**, Figure S13. Comparison of FT-IR spectra of compounds **12a–e**, Figure S14: Hue histograms (b), (d), and (f) created from fluorescent images (a), (c), and (e) of the beads **12a** (3 pieces), respectively., Figure S15. Hue histograms (b), (d), and (f) created from fluorescent images (a), (c), and (e) of the beads **12b** (3 pieces), respectively., Figure S16: Time-dependent absorption spectra when (a) beads **11**, (b) beads **12a,** (c) beads **12b** were immersed in CPC aqueous solution (200 μM), respectively.
