# Supplementary material for: Alginic Acid Beads Containing Fluorescent Solvatochromic Dyes Display an Emission Color Response to a Cationic Surfactant
Source: Polymers (Basel). 2022 Nov 1;14(21):4649. doi: 10.3390/polym14214649 (PMC9655048; doi:10.3390/polym14214649)
Supplement: Supplementary file 1 [file polymers-14-04649-s001.zip › polymers-1988880-supplementary.pdf]

Alginic acid beads containing fluorescent solvatochromic dyes display an emission color response to a cationic surfactant

Kazuki Kishi, Amane Ichimura, Zhang Shuai, Yu Otsuka, Tatsuya Morozumi, Koji Yamada

1. Synthesis

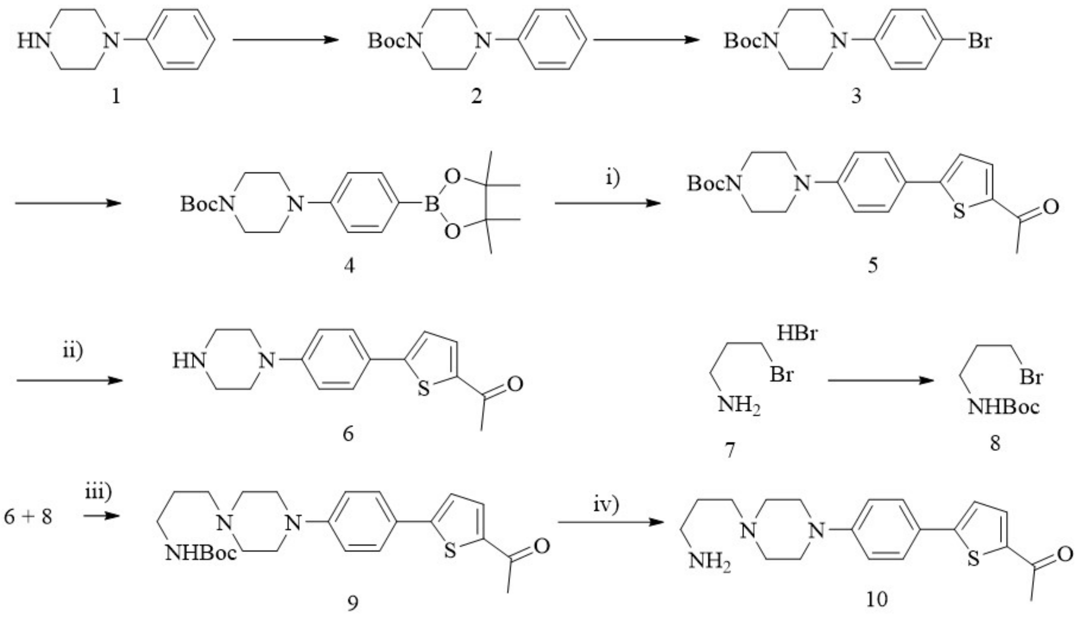

***tert*-Butyl 4-phenylpiperazine-1-carboxylate** **2**: See Reference 1.

***tert*-Butyl 4-(4-bromophenyl)piperazine-1-carboxylate** **3**: See Reference 1.

***tert*-butyl 4-(4-(4,4,5,5-tetramethyl-1,3,2-dioxaborolan-2-yl)phenyl)piperazine-1-carboxylate** **4**: See Reference 1.

***tert*-Butyl 4-(4-(5-acetylthiophen-2-yl)phenyl)piperazine-1-carboxylate** **5**: The mixture of **4** (4.5 g, 12 mmol), 2-Acetyl-5-bromothiophene (7.3 g, 35 mmol), potassium carbonate (6.5 g, 47 mmol), and tetrakis(triphenylphosphine)palladium (0) (0.41 mg, 0.35 μmol) were dissolved in *N*,*N*-dimethylformamide (30 mL) under N_2. The mixture was heated at 70 °C for overnight. The reaction was quenched by the addition of H_2O (30 mL). The solution was extracted 3 times with hexane: ethyl acetate = 4: 1 (30 mL). The organic layer was washed with $NaHCO_3$ (30 mL) and brine (30 mL) then dried over Na_2SO_4 for about 1 hour. Thereafter, Na_2SO_4 was filtrated, and the organic solvent was removed under the reduced pressure. The residue was purified by silica gel column chromatography (hexane-chloroform mixture). The product was obtained as a yellow solid (4.0 g, 11 mmol, 92 %). ^{1}H NMR (400 MHz, $CDCl_3$) δ (ppm) 7.62 (d, 1H, *J* = 3.9 Hz), 7.56 (d, 2H, *J* = 8.8 Hz), 7.20 (d, 1H, *J* = 3.9 Hz), 6.92 (d, 2H, *J* = 8.8 Hz), 3.59 (t, 4H, *J* = 10.3 Hz), 3.22 (t, 4H, *J* = 9.8 Hz) , 2.54 (s, 3H), 1.49 (s, 9H); ^{13}C NMR (100 MHz, $CDCl_3$) δ (ppm) 26.3, 28.3, 43.3, 48.3, 79.9, 115.9, 122.2, 124.5, 127.2, 133.6, 141.5, 151.4, 153.2, 154.6, 180.3; ESI–HRMS (m/z) $[M+H]^+$ calcd. for $C_{21}H_{26}O_3N_2NaS$: 409.11568 ; found: 409.11563.

1-(5-(4-(piperazin-1-yl)phenyl)thiophen-2-yl)ethan-1-one **6**: Compound **5** (2.5 g, 6.8 mmol) was dissolved in dichloromethane (50 mL). To the solution, CF_3COOH (5.2 mL, 68 mmol) was dripped carefully. The mixture was stirred for overnight at room temperature. The reaction mixture was neutralized by an aqueous solution of $NaHCO_3$. The mixed solution was extracted with chloroform (100 mL ×3). The organic layers were washed with brine (100 mL) and dried over $MgSO_4$. The organic layer was filtered with cerite and concentrated. The resulting solid was dried overnight under vacuum and used for the next step without further purification. The product was quantitatively obtained as a yellow solid. ^{1}H NMR (400 MHz, $CDCl_3$) δ (ppm) 7.62(d, 1H, *J* = 5.3 Hz), 7.55 (m,

2H), 7.19 (d, 1H, *J* = 5.38Hz), 6.92 (m, 2H), 3.22 (t, 4H, *J*=13.3Hz), 3.04 (t, 4H, *J* = 13.20Hz), 2.54 (s, 3H); ^{13}C NMR (100 MHz, DMSO-*d6*) δ (ppm) 26.3, 45.4, 48.4, 114.9, 122.5, 122.7, 127.0, 135.5, 140.7, 152.0, 152.7, 190.3; ESI–HRMS (m/z) $[M+H]^+$ calcd. for $C_{16}H_{19}N_2OS$: 287.12069 ; found: 287.12126.

***tert-Butyl (3-bromopropyl)carbamate* 8**: See Reference 2.

***tert-Butyl (3-(4-(4-(5-acetylthiophen-2-yl)phenyl)piperazin-1-yl)propyl)carbamate* 9**: Compound **6** (1.4 g, 4.9 mmol) was dissolved in tetrahydrofuran (20 mL) at 0 °C. To the solution, NaH in Oil (50~72 %, 0.24 g, 5.4 mmol) was added. The solution contains **8** (1.5 g, 5.4 mmol) in THF (10 mL) was slowly added dropwise. The temperature of the mixed solution was maintained on room temperature using ice bath for overnight. This mixed solution was quenched with water, and extracted with ethyl acetate (30 mL ×3). The organic layer was washed with brine (100 mL) and dried over $MgSO_4$. The organic layer was filtered with cerite and concentrated. The residue was purified by silica gel column chromatography (hexane-chloroform mixture). The product was obtained as a yellow solid (1.4 g, 3.1 mmol, 64 %). ^{1}H NMR (400 MHz, $CDCl_3$) δ (ppm) 7.62 (d, 1H, *J* = 3.9 Hz), 7.55 (d, 2H, *J* = 8.8 Hz), 7.19 (d, 1H, *J* = 3.9 Hz), 6.91 (d, 2H, *J* = 8.8 Hz), 3.26 (m, 6H), 2.60 (t, 4H, *J* = 9.8 Hz), 2.54 (s, 3H), 2.47 (t, 2H, *J* = 13.2 Hz), 1.71(t, 2H, *J* = 13.18 Hz), 1.44 (s, 9H); ^{13}C NMR (100 MHz, $CDCl_3$) δ (ppm) 26.3, 28.4, 39.7, 48.2, 49.0, 52.9, 53.1, 56.6, 78.8, 115.3, 115.9, 119.6, 122.0, 124.0, 127.1, 129.0, 133.7, 141.3 151.2, 151.5, 153.4, 156.0, 190.3, ; ESI–HRMS (m/z) $[M+H]^+$ calcd for $C_{24}H_{34}O_3N_3S$: calcd. 444. 23122 ; found: 444.23154.

***1-(5-(4-(4-(3-aminopropyl)piperazin-1-yl)phenyl)thiophen-2-yl)ethan-1-one* 10:** Compound **9** (1.4 g, 3.1mmol) was dissolved in dichloromethane (50 mL). Trifluoroacetic acid (2.4 mL, 0.13 mol) was slowly dripping into the solution. After the addition reaction was carried out overnight at room temperature. The mixture was neutralized by adding the aqueous solution of $NaHCO_3$. The mixed solution was extracted with chloroform (100 mL × 3). The organic layer was washed with brine (100 mL) and dried over $MgSO_4$ and concentrated. The resulting solid was dried under vacuum, and used for the next step without further purification. The product was obtained as a yellow solid (0.99 g, 2.9 mmol, 92 %). ^{1}H NMR (400 MHz, $CDCl_3$) δ (ppm) 7.62 (d, 1H, J=3.9 Hz), 7.54 (d, 2H, *J*=16.1Hz), 7.19 (d, 1H, *J* = 3.9 Hz), 6.92 (d, 2H, *J* = 8.8 Hz), 3.28 (m, 6H), 2.63 (m, 4H), 2.54 (s, 3H), 2.47 (m, 2H, *J* = 7.32Hz), 1.88 (m, 2H) ; ESI–HRMS (m/z) [M+H]+ calcd for $C_{19}H_{26}N_3OS$: 344.17851 ; found: 344.17911.

Alginic acid beads containing fluorescent solvatochromic dyes
display an emission color response to a cationic surfactant
Kazuki Kishi, Amane Ichimura, Zhang Shuai, Yu Otsuka, Tatsuya Morozumi, Koji Yamada

2. NMR and Mass measurement results

2. 1. Compound **5**

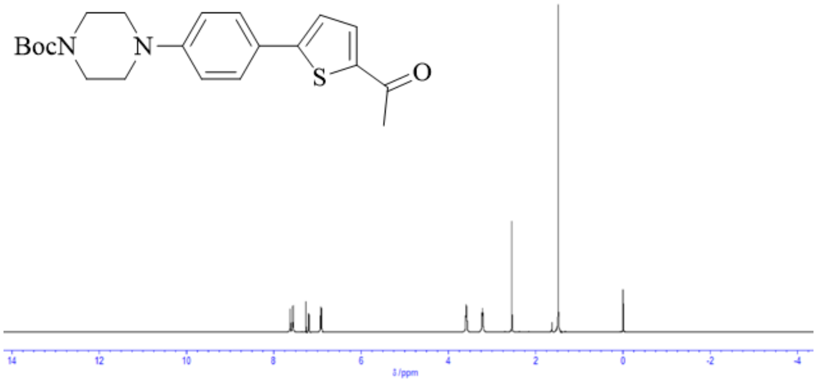

Figure S1. ^{1}H NMR spectrum of **5** measured in $CDCl_3$.

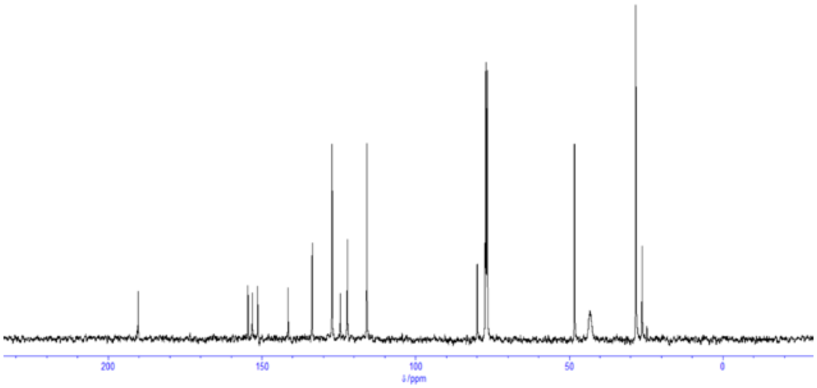

Figure S2.^{13}C NMR spectrum of **5** measured in $CDCl_3$.

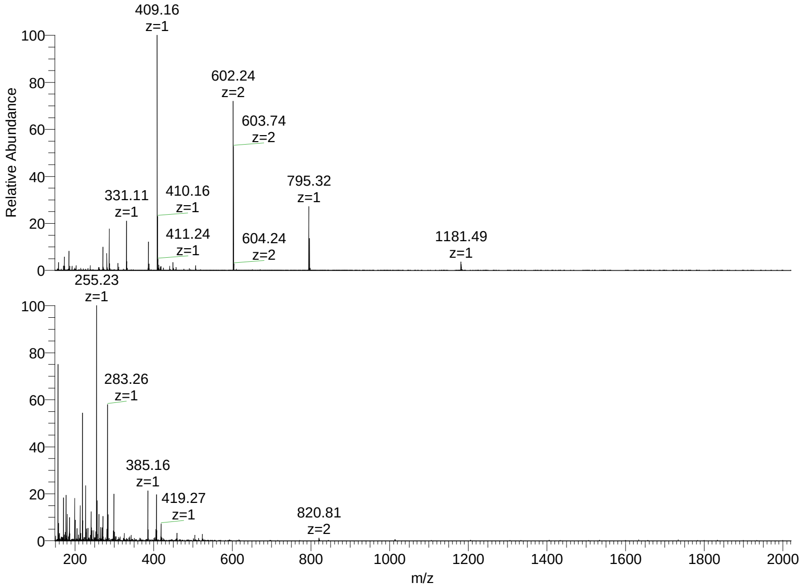

Figure S3. ESI-MS (m/z) of **5**.

2. 2. Compound **6**

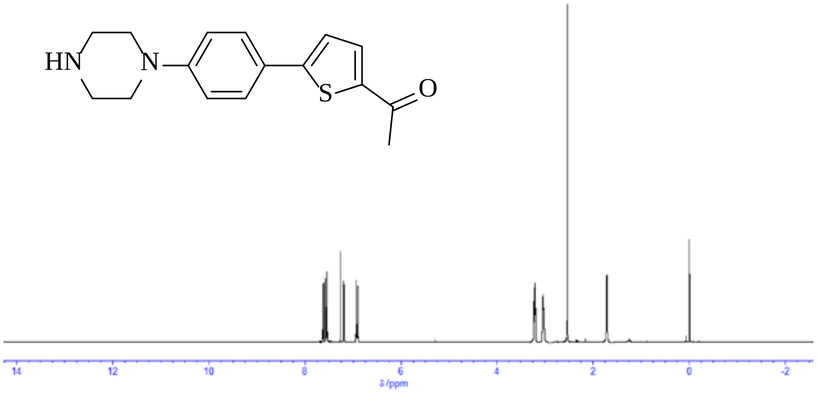

Figure S4. ^{1}H NMR spectrum of **6** measured in $CDCl_3$.

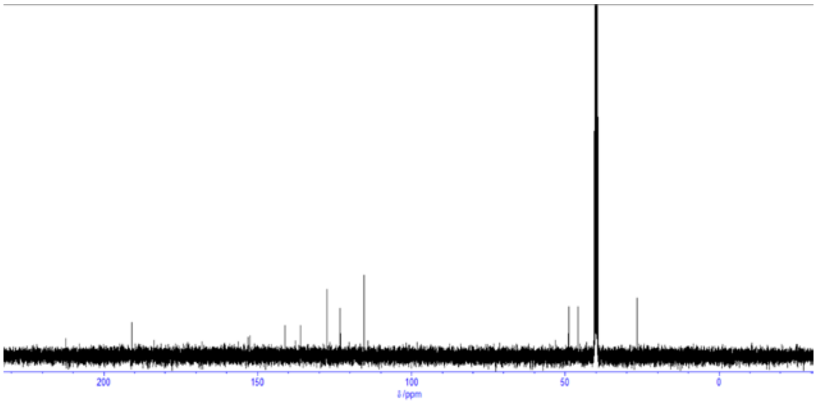

Figure S5. ^{13}C NMR spectrum of **6** measured in DMSO.

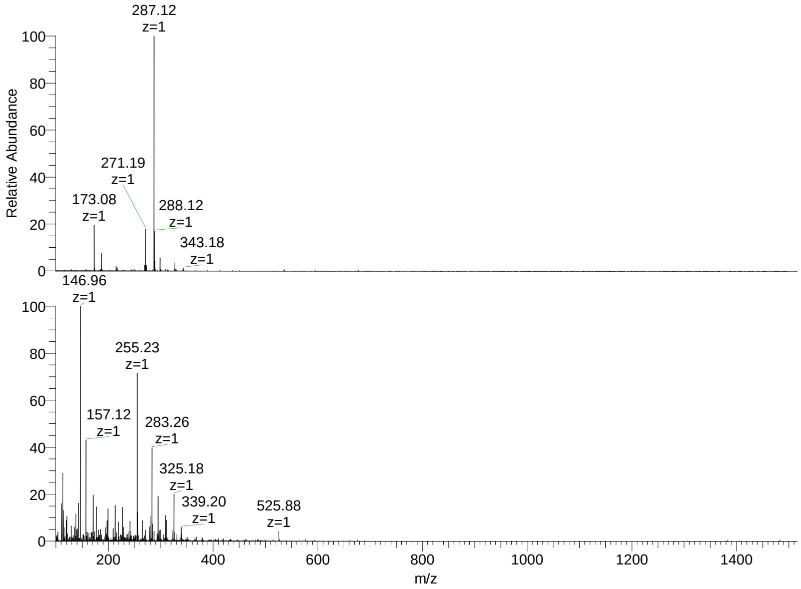

Figure S6. ESI-MS (m/z) of **6**.

2. 3. Compound **9**

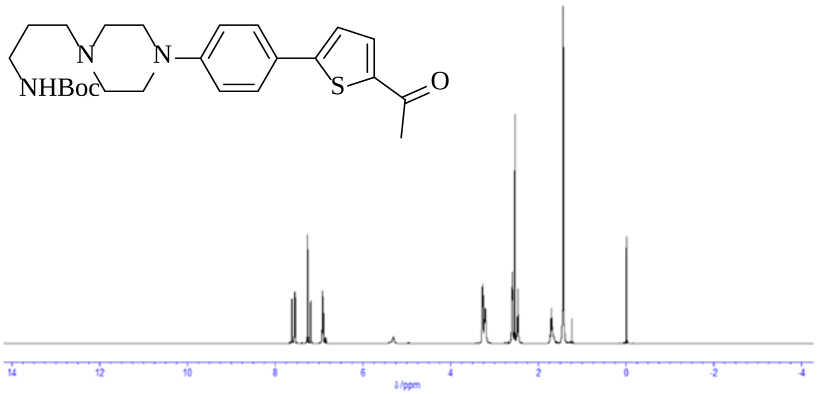

Figure S7. ^{1}H NMR spectrum of **9** measured in $CDCl_3$.

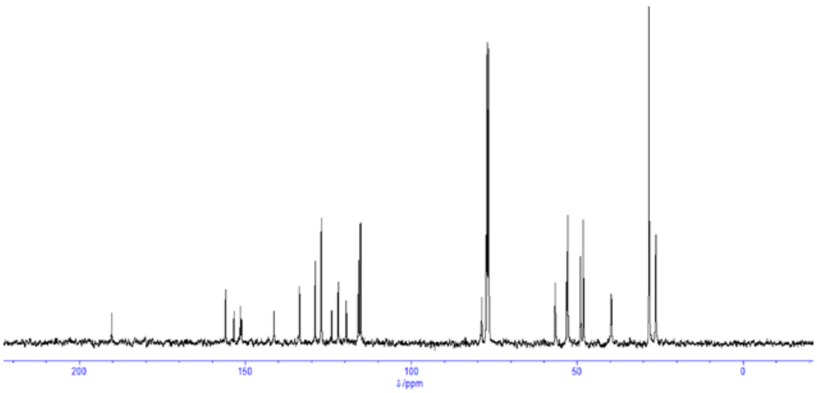

Figure S8. ^{13}C NMR spectrum of **9** measured in $CDCl_3$.

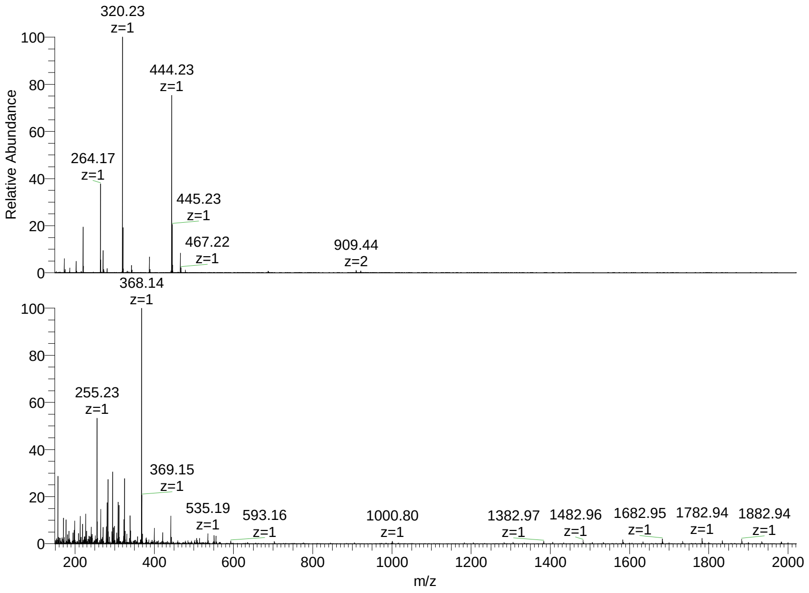

Figure S9. ESI-MS (m/z) of **9**.

2. 4. Compound **10**

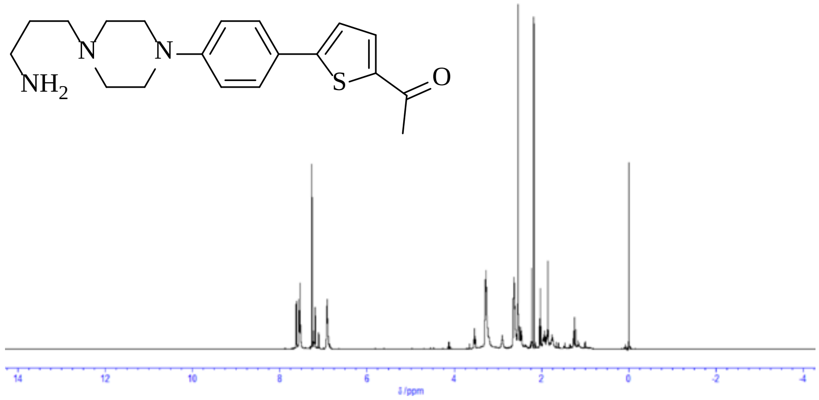

Figure S10. ^{1}H NMR spectrum of **10** measured in $CDCl_3$.

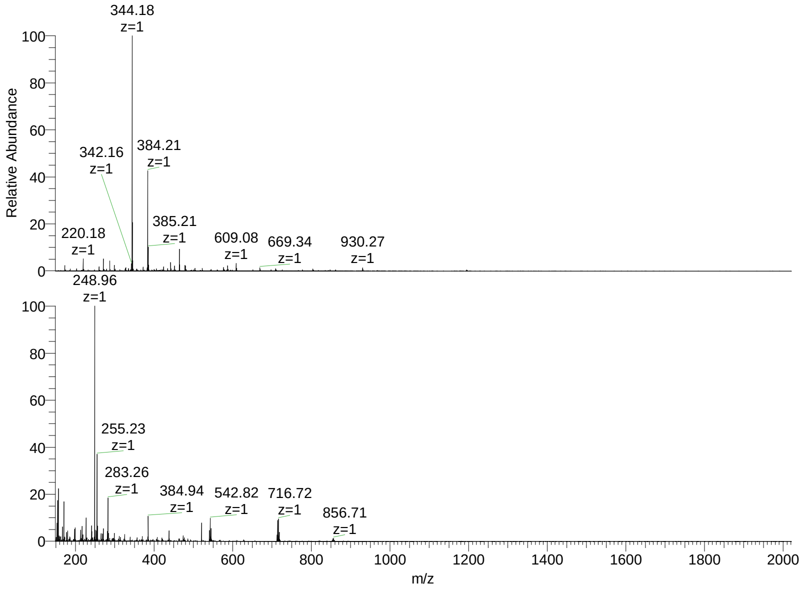

Figure S11. ESI-MS (m/z) of **10**.

3. FT-IR measurement results

3. 1. FT-IR spectra of compounds **11** and **12a** ~ **12e**

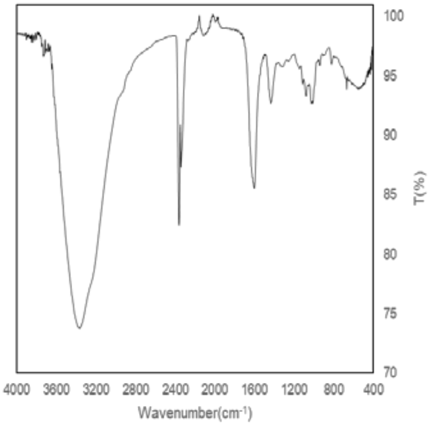

(**a**)

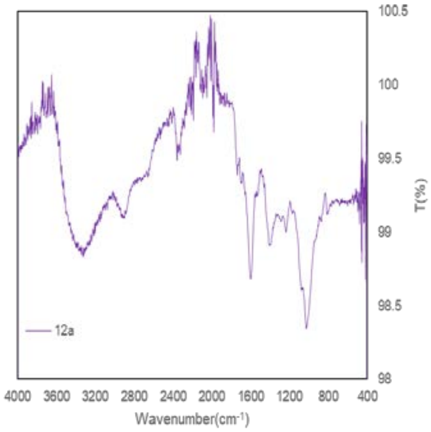

(**b**)

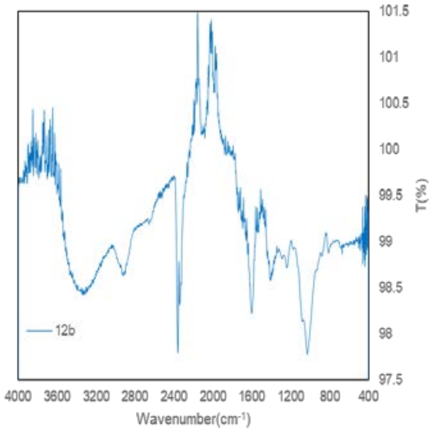

(**c**)

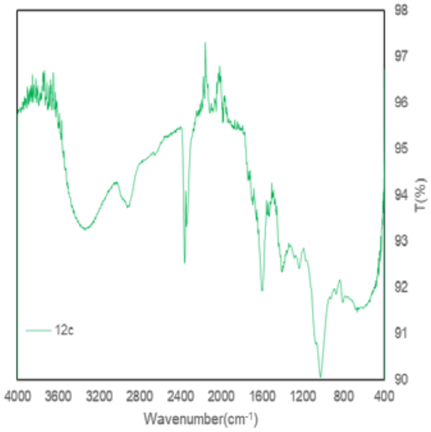

(**d**)

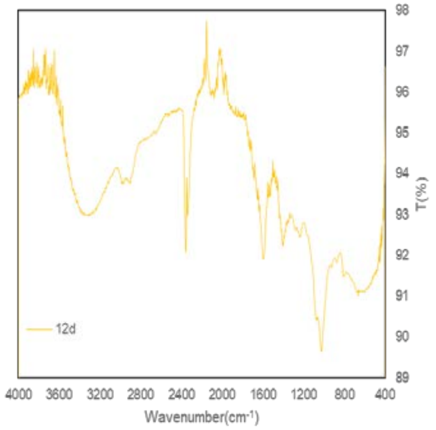

(e)

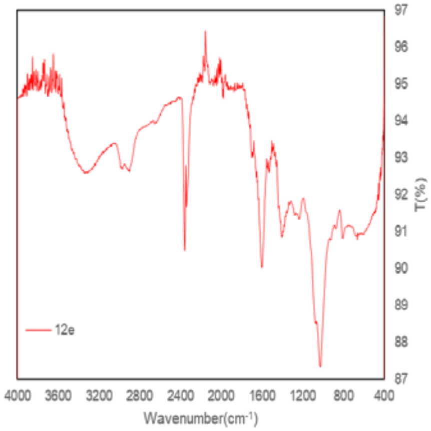

(**f**)

Figure S12. FT-IR spectra of (**a**) **11**, (**b**) **12a**, (**c**) **12b**, (**d**) **12c**, (e) **12d**, and **(f) 12e**.

3. 2. Comparison of FT-IR spectra of compounds **12a** ~ **12e**

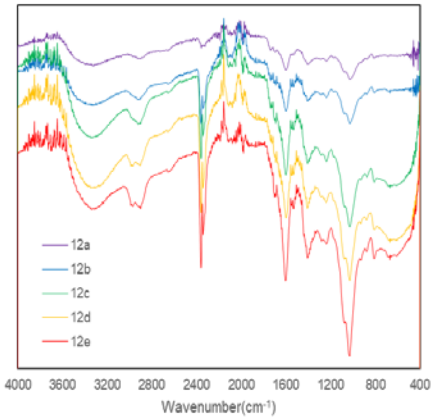

Figure S13. Comparison of FT-IR spectra of compounds **12a** ~ **12e**.

4. Fluorescent images and hue histograms of beads

4-1. Fluorescent images and hue histograms of beads **12a** (3 pieces)

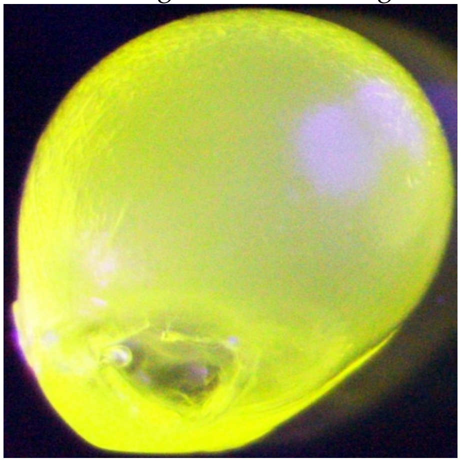

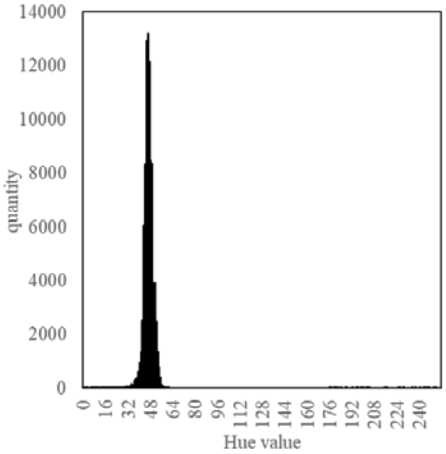

(**a**) (**b**)

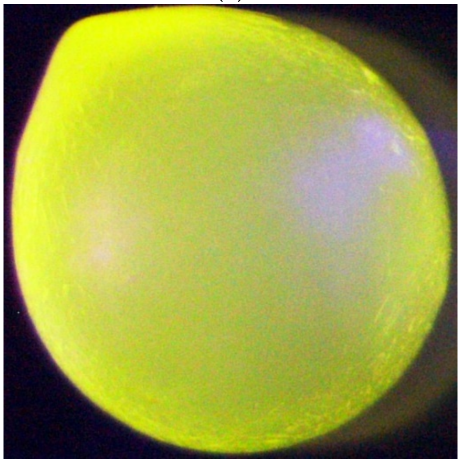

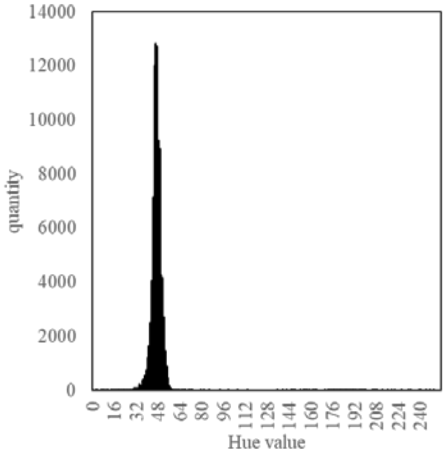

(**c**) (**d**)

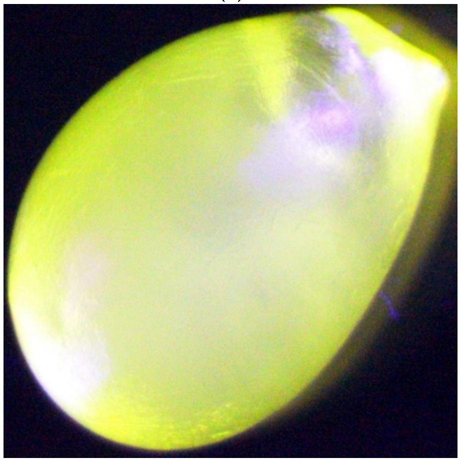

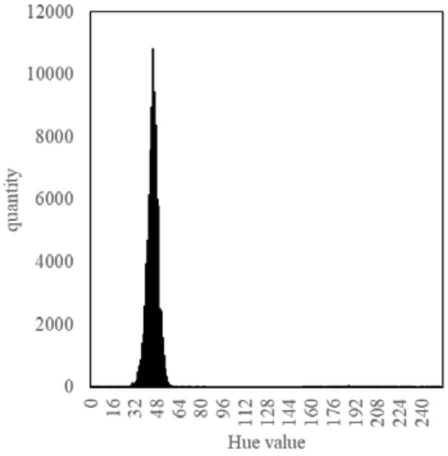

(**e**) (**f**)

Figure S14. Hue histograms (b), (d), and (f) created from fluorescent images (a), (c), and (e) of the beads **12a** (3 pieces), respectively.

4-2. Fluorescent images and hue histograms of beads **12b** (3 pieces)

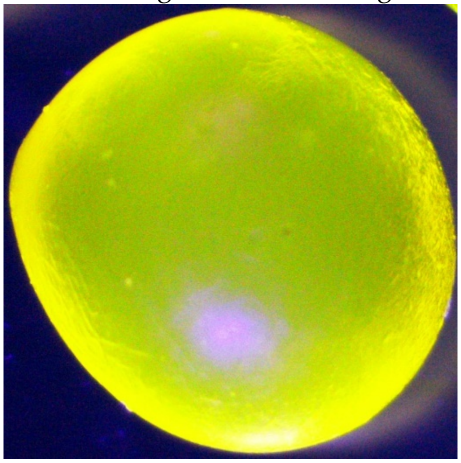

(**a**)

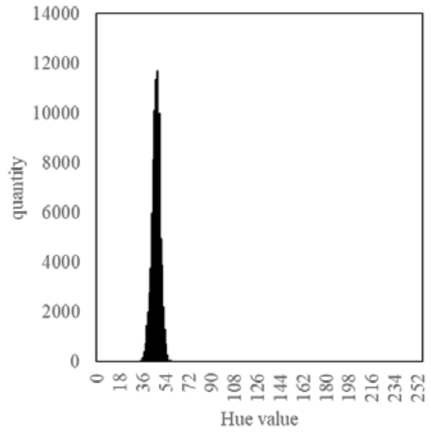

(**b**)

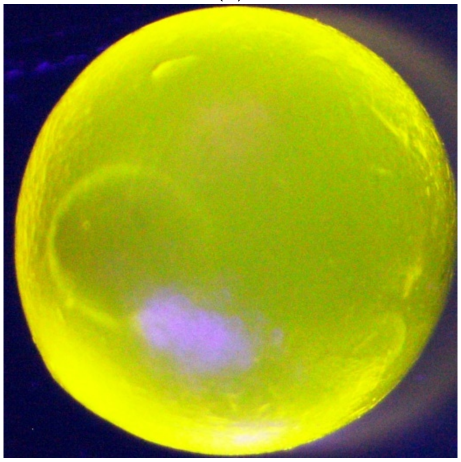

(**c**)

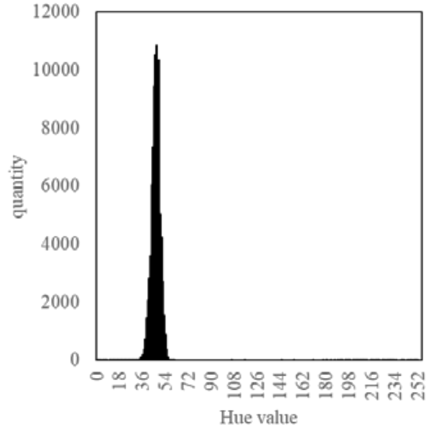

(**d**)

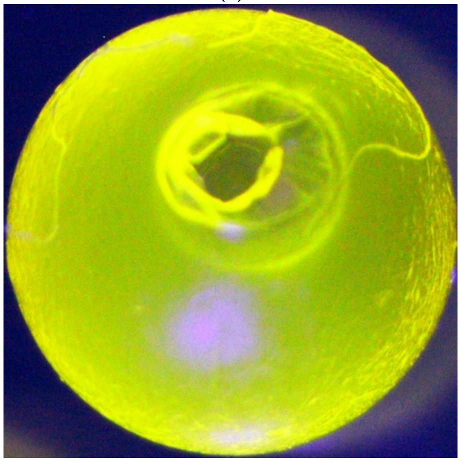

(**e**)

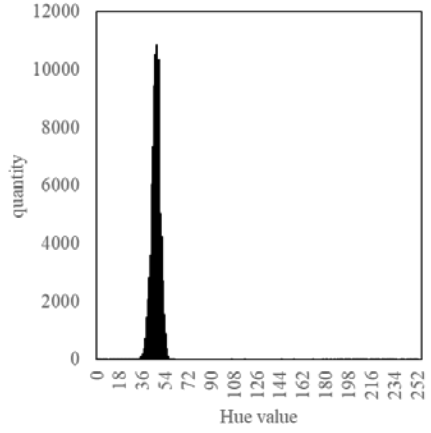

(**f**)

Figure S15. Hue histograms (b), (d), and (f) created from fluorescent images (a), (c), and (e) of the beads **12b** (3 pieces), respectively.

5. Time-dependent absorption spectra of CPC solution

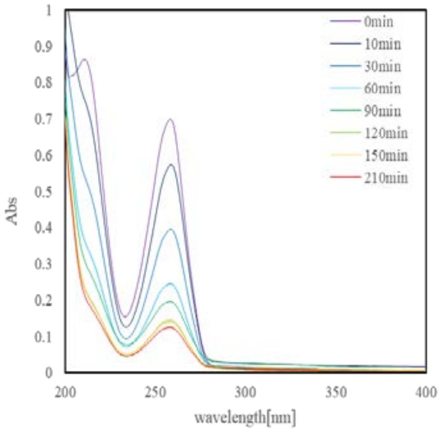

(**a**)

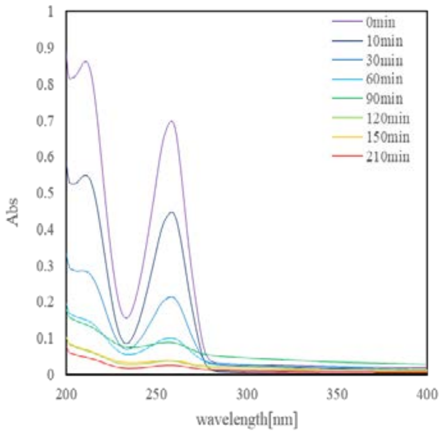

(**b**)

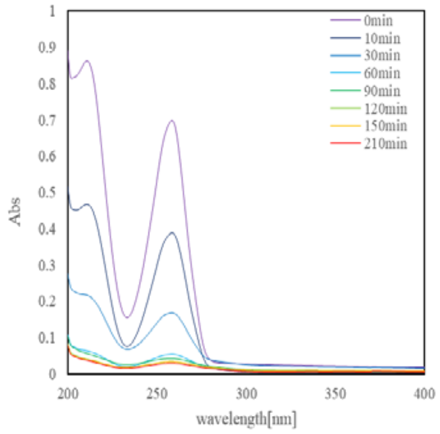

(**c**)

Figure S16. Time-dependent absorption spectra when (a) beads **11**, (b) beads **12a,** (c) beads **12b** were immersed in CPC aqueous solution (200 μM), respectively.

6. References

[1] Otsuka, Y. Synthesis of fluorescent solvatochromic beads via Suzuki-Miyaura cross-coupling on the surface and its optical waveguide spectra to fabrication of bio-affinity sensing device. Sapporo, Hokkaido University, **2021**, *Ph. D. thesis*.

[2] Levy, D. E. et al., Aryl–indolyl maleimides as inhibitors of CaMKIIδ. Part 1: SAR of the aryl region, *Bioorganic & Medicinal Chemistry Letters*. **2008**, 18, 2390–2394.
